# Effective Oxygen Reduction Reaction Performance of FeCo Alloys In Situ Anchored on Nitrogen-Doped Carbon by the Microwave-Assistant Carbon Bath Method and Subsequent Plasma Etching

**DOI:** 10.3390/nano9091284

**Published:** 2019-09-08

**Authors:** Mincong Liu, Feng Yu, Cunhua Ma, Xueyan Xue, Haihai Fu, Huifang Yuan, Shengchao Yang, Gang Wang, Xuhong Guo, Lili Zhang

**Affiliations:** 1 Key Laboratory for Green Processing of Chemical Engineering of Xinjiang Bingtuan, School of Chemistry and Chemical Engineering, Shihezi University, Shihezi 832003, China (M.L.) (C.M.) (X.X.) (H.F.) (H.Y.) (S.Y.) (G.W.); 2 State Key Laboratory of Chemical Engineering, East China University of Science and Technology, Shanghai 200237, China; 3 Institute of Chemical and Engineering Sciences, Agency for Science, Technology and Research, Jurong Island 627833, Singapore

**Keywords:** FeCo alloy, oxygen reduction reaction, microwave-assisted carbon bath method, plasma, defect sites

## Abstract

Electrocatalysts with strong stability and high electrocatalytic activity have received increasing interest for oxygen reduction reactions (ORRs) in the cathodes of energy storage and conversion devices, such as fuel cells and metal-air batteries. However, there are still several bottleneck problems concerning stability, efficiency, and cost, which prevent the development of ORR catalysts. Herein, we prepared bimetal FeCo alloy nanoparticles wrapped in Nitrogen (N)-doped graphitic carbon, using Co-Fe Prussian blue analogs (Co_3_[Fe(CN)_6_]_2_, Co-Fe PBA) by the microwave-assisted carbon bath method (MW-CBM) as a precursor, followed by dielectric barrier discharge (DBD) plasma treatment. This novel preparation strategy not only possessed a fast synthesis rate by MW-CBM, but also caused an increase in defect sites by DBD plasma treatment. It is believed that the co-existence of Fe/Co-N sites, rich active sites, core-shell structure, and FeCo alloys could jointly enhance the catalytic activity of ORRs. The obtained catalyst exhibited a positive half-wave potential of 0.88 V vs. reversible hydrogen electrode (RHE) and an onset potential of 0.95 V vs. RHE for ORRs. The catalyst showed a higher selectivity and long-term stability than Pt/C towards ORR in alkaline media.

## 1. Introduction

Fuel cells and rechargeable zinc-air batteries are the most promising clean auto power for the next generation due to their low cost and high energy density [1,2,3,4]. It is already widely known that the oxygen reduction reaction (ORR) is a slow kinetic process in cathodic reactions [5,6]. Precious metal Pt-based electrocatalysts usually possess high catalytic activity for ORRs, while their application for ORRs is not satisfactory, because of the problems, such as high cost and poor long-term durability [7,8]. Therefore, it is essential to develop electrocatalysts with high activity, long life, and low cost to substitute for the conventional catalysts of ORRs [9,10,11].

In order to reduce the loading of Pt and Pt-M (M = Fe, Co, Ni, Cu etc.), alloy catalysts have been extensively discussed [12,13,14]. The second element alloyed with Pt can adjust the catalytic ability of the surface of the Pt-based catalyst and improve the catalytic performance [15]. For example, Zhang et al. [16] synthesized bimetallic PtNi/C with hollow structures through a facile solution-based approach and observed distance lattice contraction of Pt due to the presence of Ni-improved ORR performance. It was found that PtNi/C showed excellent half-wave potential (E_1/2_) of 0.88 V vs. reversible hydrogen electrode (RHE) and a stable 4-electron pathway. At the meantime, the morphology and size of Pt-M alloys also have an important influence on the electrocatalytic performance. Ma et al. [17] used a simple method to synthesize a hexagonal nanosheet PtFe alloy, a hexagonal nanosheet PtFe alloy with uniform distribution and ultra-small (ca. 2.6 nm) particle size, as a high efficiency electrocatalyst. The PtFe alloy showed the highest initial potential of 0.95 V vs. RHE. Up till now, non-Pt of the M_1_-M_2_ (M_1_, M_2_ = Fe, Co, Ni etc.) system has been reported, which is beneficial to improve the conductivity of catalysts and activate each other’s active sites by the doping of M_1_/M_2_ [18,19]. Wen et al. [20] reported FeCo alloy nanoparticles embedded in Nitrogen (N)-doped carbon with excellent ORR performance, which was attributed to the core-shell nanostructure, the large specific surface area, and the synergetic effect of the mutual element for FeCo@NC. Wang et al. [21] prepared FeNi nanoparticles wrapped in N-doped carbon nanotubes (NCNTS,) in which NCNTS effectively prevented the oxidation and aggregation of FeNi nanoparticles, showing an initial potential of 0.95 V vs. RHE. All the previous research showed that the catalysts with bimetallic active sites exhibited optimal performance with large surface areas, porous nanostructures, and rich active centers.

Herein, we prepared FeCo alloy nanoparticles wrapped with N-doped carbon (FeCo@NC) using Co-Fe PBA as a precursor via the microwave-assisted carbon bath method (MW-CBM). Dielectric barrier discharge (DBD) plasma is then used to produce the catalyst with more defect sites (DBD-FeCo@NC). PBA have different bimetallic compositions, uniform sizes, morphology, and structure, which are considered to be ideal precursors for the synthesis of hollow and porous electrocatalysts [22]. In addition, MW-CBM has been successfully used in our previous work of LiFePO_4_/C [23], LFePO_4_/MEGO [24], Fe/C [25], and Ni/VMT [26]. The MW-CBD has rapid heating efficiency due to the high efficiency of microwave absorption of columnar carbon. due to its advantages of rapid heating efficiency and low side reactions [27]. The MW-CBD prevents the reaction between air and catalyst precursor due to the air reacting with the columnar carbon during the heating process. Moreover, the plasma-assisted preparation method is used in the synthesis and modification of electrocatalyst materials, such as making active sites or exfoliating catalysts [28,29]. The as-prepared DBD-FeCo@NC exhibited good electrochemical ORR performance, e.g., a positive half-wave potential of 0.88 V vs. RHE and an onset potential of 0.95 V vs. RHE. We believe that this strategy provides potential for the preparation of similar superstructures of other effective catalysts with much active sites.

## 2. Materials and Methods

### 2.1. Synthesis of Samples

Synthesis of Co_3_[Fe(CN)_6_]_2_ (Co-Fe PBA): 2 mmol of K_3_[Fe(CN)_6_] was dissolved in 100 mL deionized (DI) water and is labelled as solution A. A total of 3 mmol of Co(NO)_2_ was dissolved in 100 mL DI water and is labeled as solution B. Solution B was slowly added to solution A under continuous magnetic stirring and the mixture was left for stirring for 3.5 h at room temperature. After aging for 24 h without stirring at room temperature, the precipitate was centrifuged for several times with DI water and absolute ethanol. The final product of Co-Fe PBA was obtained after drying at 80 °C for 8 h under vacuum.

Synthesis of FeCo@NC catalysts: 1.0 g of Co-Fe PBA was put into a small graphite crucible (d = 1 cm, h = 1.5 cm). The small graphite crucible containing Co-Fe PBA was then put inside a 150 mL crucible and the graphite crucible was embedded in commercial columnar carbon material. The 150 mL crucible with a small graphite crucible inside was then placed in a commercial microwave oven under microwave irradiation with 900 W for 10 min. The obtained product was further treated by 0.5 M H_2_SO_4_ with ultrasound for 1 h to remove impurities. The product was washed by centrifugation using DI water. The final product was obtained after drying at 80 °C in a vacuum and is denoted as FeCo@NC.

Synthesis of DBD-FeCo@NC catalysts: 50 mg of FeCo@NC was treated in Ar atmosphere under a DBD plasma reactor at an input power of 50 V × 1.5 A AC (alternating current) for 30 min to prepare DBD-FeCo@NC.

### 2.2. Characterizations

The field emission Tecnai G2 F20 electron (Hillsboro, OR, USA) microscope was used to analyze transmission electron microscopy (TEM). X-ray diffraction (XRD, D8 Advance, Bruker, Karlsruhe, Germany) with Cu-K radiation was used to characterize the structures of crystallographic phases for the products. The Raman spectra was analyzed by a Laser Confocal Micro-Raman Spectroscope (LabRAM HR800, Horiba Jobin Yvon, French) with a laser wavelength of 532 nm. The surface chemical compositions were tested by using an X-ray photoelectron spectroscope (XPS, ESCALAB 250Xi, Thermo Fisher Scientific, MA, USA).

### 2.3. Electrochemical Measurements

#### 2.3.1. ORR Text

The electrochemical performance was tested by using a CHI760D electrochemical station with a three-electrode cell system at room temperature in 0.1 M KOH (Potassium hydroxide) as an electrode solution. The reference electrode and the counter electrode was the Ag/AgCl electrode and a Pt wire, respectively. To prepare the catalyst ink for electrochemical analysis, 5 mg of the catalyst was dispersed into 0.5 mL of ethanol containing a Nafion solution (5 wt%, DuPont) with the aid of ultrasonication. A total of 10 μL of the catalyst ink was then coated on the glassy carbon disc electrode (3 mm in diameter) and dried at 60 °C. The catalyst loading was controlled at 0.0142 mg/cm^2^. The catalyst on the glassy carbon rotating disk electrode was used as a working electrode, with a rotating rate varying from 625 to 2500 rpm at a scan rate of 10 mV/s. Ag/AgCl and platinum plate were used as the reference and counter electrodes, respectively. The cyclic voltammetry (CV) curves were obtained at a sweep speed of 50 mV/s in the potential range between −0.8 and 0.2 V after purging O_2_ or N_2_ for 20 min.

In a typical ORR program, the following equation can be used to estimate the number of electron transfers based on the slope of the Koutecky–Levich (K–L) graph:

(1) $$\frac{1}{J}=\frac{1}{J_{k}}+\frac{1}{B\omega^{1/2}}$$

where *J* represents the current density measured on a rotating disk electrode (RDE), *J_k_* is the kinetic current density, and *ω* acts as the electrode rotation speed. *B* is derived from the following equation:

(2) $$B=0.2nFC_{0}D_{0}^{2/3}\nu^{-1/6}$$

among which *n* represents the electron transfer number of each O_2_ molecule in the ORR process, *F* is 96,485 C/mol (Faraday constant), *C*_0_ is 1.2 × 10^−3^ mol/L (the dissolved O_2_ concentration), *D*_0_ is 1.9 × 10^−5^ cm^2^/s (the O_2_ diffusion coefficient), and *ν* is 0.01 cm^2^/s (the electrolyte kinematic viscosity).

#### 2.3.2. OER (Oxygen Evolution Reaction) Text

The electrochemical performances of the prepared products were measured in an O_2_-saturated 1 M KOH at a 10 mV/s scanning rate with a three-electrode system using a CHI760D electrochemical station. The catalyst ink coated on Ni-foam was used as the working electrode. Pt foil and Ag/AgCl were used as the counter electrode and reference electrode, respectively. To prepare the catalyst ink, 2 mg of samples were dispersed in 500 μL water and 500 μL ethanol mixture, which contained 30 mL 60 wt% polytetrafluoroethylene (PTFE) solution with the aid of ultrasonication. Subsequently, 100 μL of as-prepared catalyst ink was loaded on surface of the Nickel foam surface (1 × 1 cm^2^) and then dried at room temperature.

All potentials by measuring were called reversible hydrogen electrode (RHE) by RHE calibration, as follows:

E_RHE_ = E_Ag/AgCl_ + 0.197 + 0.059 pH (3)

For the whole polarization curve, linear sweep voltammetry (LSV) was performed at a 1.0 mV/s scanning rate, which were IR (deviation caused by I-current and R-resistance) corrected. Calculating the overpotential (*η*) is expressed as follows: *η* = E_RHE_ − 1.23 V.

## 3. Results

**Figure 1:**
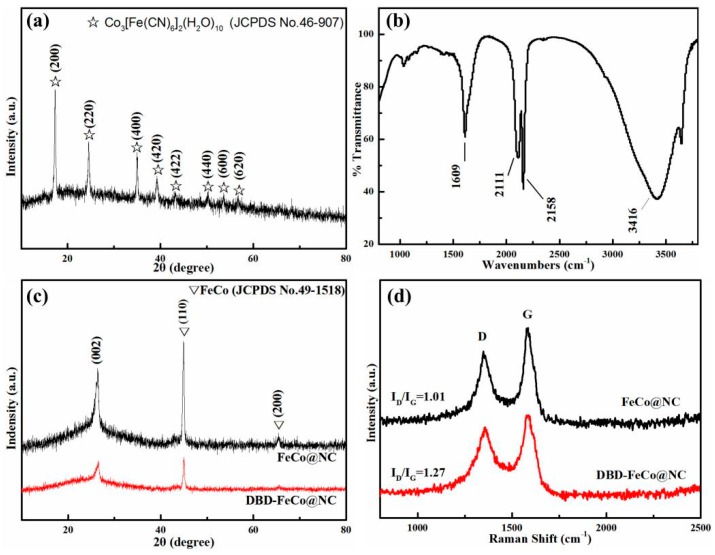
(**a**) XRD pattern, (**b**) FTIR spectra of CoFe-PBA, (**c**) XRD pattern, and (**d**) Raman spectra of FeCo@NC and DBD-FeCo@NC.

As shown in Figure 1a, it was observed from the XRD pattern of Co-Fe PBA that the peaks of XRD are related to the Co_3_[Fe(CN)_6_]_2_(H_2_O)_10_ (JCPDS No. 46-907) [20]. At the same time, Figure 1b showed the FTIR spectrum of CoFe-PBA. It can be seen that Fe^III^-CN-Co^II^ and Fe^II^-CN-Co^III^ appeared at positions of 2111 cm^−1^ and 2158 cm^−1^, respectively. Additionally, two peaks at 1609 cm^−1^ and 3416 cm^−1^ corresponding to the bending vibration and stretching vibration absorption peak of O-H in water molecules were obviously detected, indicating that some water molecules entered the PBA lattice. Combined with the XRD spectrum and FTIR spectrum, it was shown that CoFe-PBA was successfully synthesized.

In order to determine the structural feature of the as-prepared products, the XRD pattern (Figure 1c) of FeCo@NC and DBD-FeCo@NC were presented in Figure 1c. The XRD patterns of the two showed a sharp peaks at 44.8°and 65.3°, which can be indexed to the diffraction from (110) and (200) planes of FeCo alloy (JCPDS No. 49-1567) [30]. Additionally, the two catalysts had broad peaks at 2θ = 26°, which formed on the (002) plane of graphite. FeCo@NC prepared by MW-CBM exhibited a high degree of graphitization. However, the peak intensity of graphite carbon was reduced for DBD-FeCo@NC after plasma treatment, indicating that the crystallinity of graphite carbon was decreased. The molecular structures of FeCo@NC and DBD-FeCo@NC were investigated using the Raman spectra. As can be seen from Figure 1d thatthe G band and the D band were around 1580 cm^−1^ and 1350 cm^−1^, respectively. The value of I_D_/I_G_ can be used to estimate the defect levels in the Raman spectra of carbon-based materials. It was found that the I_D_/I_G_ values were 1.01 for FeCo@NC, and 1.27 for DBD-FeCo@NC, respectively, indicating that the plasma treatment promoted the defective sites for DBD-FeCo@NC [31,32]. This conclusion was consistent with our XRD results and high-resolution TEM (HRTEM) images. The catalysts exposed more active sites due to increased defect sites, which was beneficial for ORR performance.

**Figure 2:**
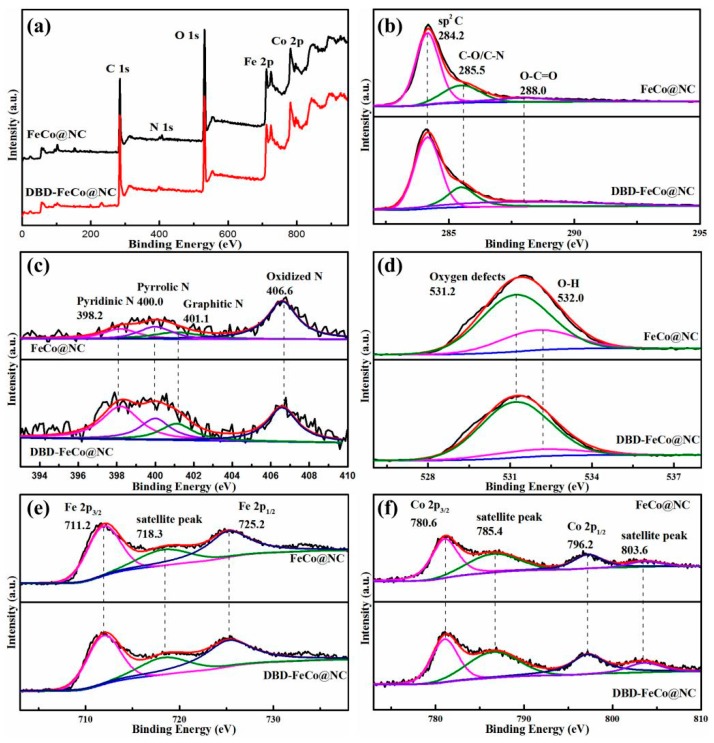
(**a**) XPS spectrum, (**b**) C 1s spectra, (**c**) N 1s spectra, (**d**) O 1s spectra, (**e**) Fe 2p spectra, (**f**) Co 2p spectra of FeCo@NC, and DBD-FeCo@NC.

XPS was further employed to survey the surface composition of the FeCo@NC and DBD-FeCo@NC. Figure 2a showed the survey XPS spectrum of FeCo@NC and DBD-FeCo@NC. Figure 2b showed that C 1s spectra can be divided into three peaks, corresponding to sp^2^ hybridized C (284.2 eV), C-O/C-N (285.5 eV), and O-C=O (288.0 eV) [33]. Figure 2c displayed the N 1s spectra of FeCo@NC and DBD-FeCo@NC, which could be deconvoluted into four peaks relevant to pyridinic N (398.2 eV), pyrrolic N (400.0 eV), graphitic N (401.1 eV), and oxidized N (406.6 eV). All of these N species were reported to show advantages for ORR, apart from the uncertain contribution of the oxidized N. As shown in Table 1, the different nitrogen type contents of FeCo@NC and DBD-FeCo@NC were 0.26 vs 0.58 at.% (pyridinic N), 0.30 vs 0.33 at.% (pyrrolic N), 0.26 vs 0.26 at.% (graphitic N), and 0.98 vs 0.50 at.% (oxidized N), respectively. It was found that DBD-FeCo@NC expressed an increased in the content of pyridinic N after plasma treatment, which was good for ORR activity [34,35]. The O 1s XPS spectra of all samples were given in Figure 2c. The peak at 531.2 eV and 532.0 eV belonged to oxygen defects and O-H sites from surface-absorbed water, respectively. Moreover, the amount of oxygen defects for FeCo@NC and DBD-FeCo@NC was 72.98% and 85.57%, respectively. The increase of oxygen vacancies after plasma treatment was beneficial to the adsorption and reduction of oxygen [36,37]. Figure 2e showed the Fe 2p spectra of FeCo@NC and DBD-FeCo@NC. The peak at the binding energy of 711.2 eV corresponded to Fe 2p_3/2_, the peak at 725.2 eV was relevant to Fe 2p_1/2_, and the peak at 718.4 eV was a satellite peak, confirming the existence of Fe-*N*-C structure [38]. The high-resolution XPS spectra of Co 2p (Figure 2f) revealed that there was a weak pair of doublets for the Co 2p_3/2_ and Co 2p_1/2_ signals at 780.6 eV and 796.2 eV, and the peak at 719.0 eV was a satellite peak, indicating the presence of Co-N-C species [39].

**Figure 3:**
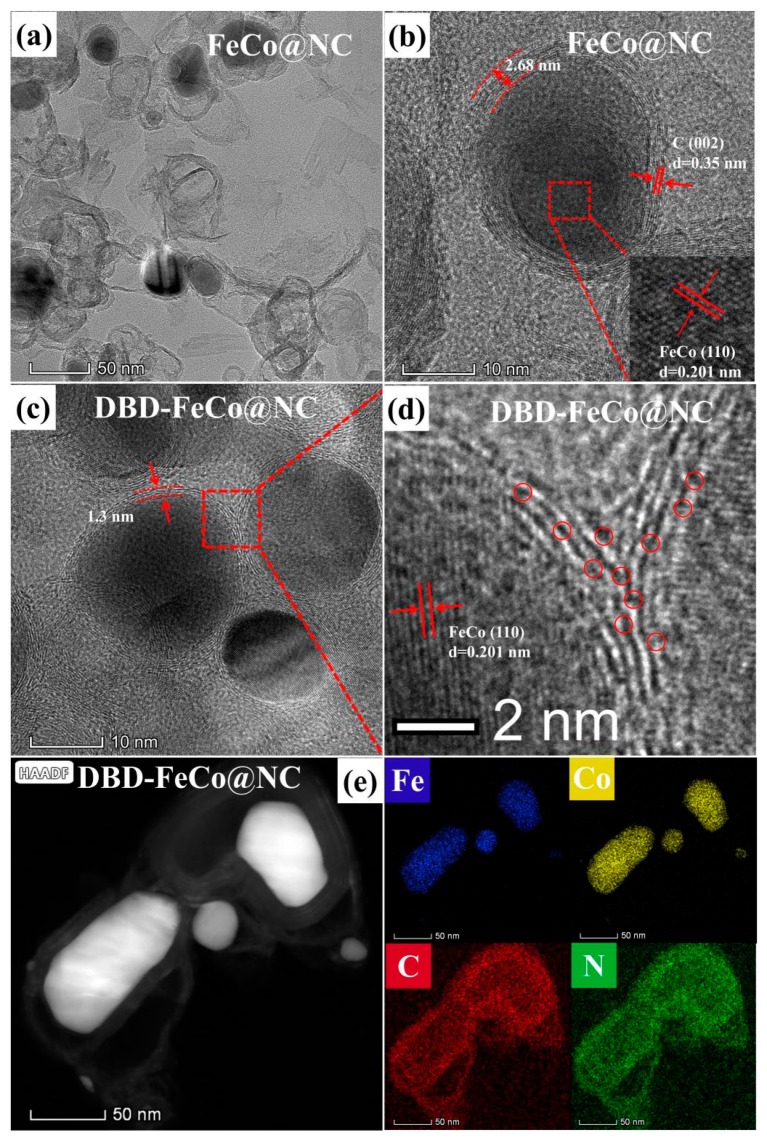
(**a**) Transmission electron microscopy (TEM) image for FeCo@NC, (**b**) high-resolution TEM (HRTEM) image for FeCo@NC, (**c**,**d**) HRTEM images for dielectric barrier discharge (DBD)-FeCo@NC, (**e**) HAADF-STEM image and the corresponding elemental mapping of DBD-FeCo@NC.

The morphology of FeCo@NC and DBD-FeCo@NC was investigated by transmission electron microscopy (TEM). A closer view of the TEM image displayed that FeCo@NC existed in a core-shell structure with a diameter of 30–50 nm (Figure 3a). The high-resolution TEM (HRTEM) in Figure 3b of FeCo@NC further revealed that the FoCo alloy nanoparticles were wrapped by graphitic carbon layers. The well-defined crystalline lattice gaps were 0.201 nm (core) and 0.35 nm (shell), which associated with the (110) plane of the FeCo phase and (002) the plane of the graphitic carbon, respectively. The HRTEM image in Figure 3c disclosed that DBD-FeCo@NC maintained a core-shell structure well after plasma treatment, and the lattice fringe spacing (0.201 nm) of the FeCo phase can also be observed. It was worth noting that the thickness of the graphite carbon layer of DBD-FeCo@NC was stripped from 2.68 nm to 1.06 nm after plasma treatment, compared to FeCo@NC. The thin layer structure of DBD-FeCo@NC reduced the surface reaction resistance of the catalyst, which was beneficial to ion transport/transfer [40]. More importantly, there were a few defects in DBD-FeCo@NC, which indicated that the interface active site was enhanced by plasma treatment and was beneficial for ORR [41,42]. HAADF-STEM (High-Angle Annular Dark Field- Scanning transmission electron microscopy) images (Figure 3e) and relevant elemental mapping of Fe, Co, C, and N revealed the core-shell morphology with C, N, Fe, and Co and the uniform distribution of Fe and Co elements in the core-shell structure, proving that the active centers were doped and distributed uniformly on the catalyst.

**Figure 4:**
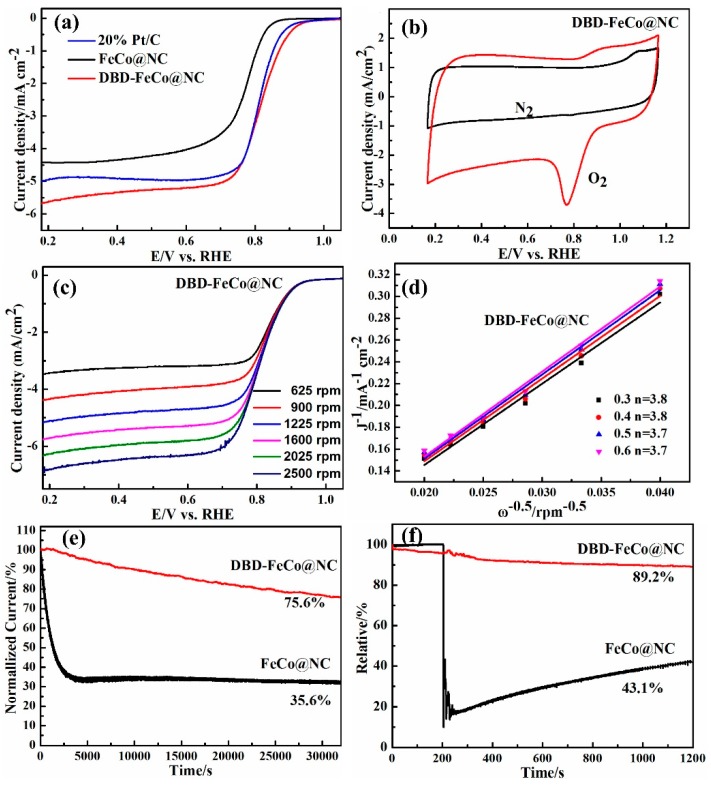
(**a**) Linear sweep voltammetry (LSV) curves of FeCo@NC, DBD-FeCo@NC, and 20 wt.% Pt/C in O_2_-saturated 0.1 M KOH, with a speed of 1600 rpm at a sweep rate of 10 mV/s; (**b**) cyclic voltammetry (CV) curves of DBD-FeCo@NC at a scan rate of 50 mV/s in N_2_-saturated or O_2_-saturated 0.1 M KOH electrolyte; (**c**) LSVs of DBD-FeCo@NC at various rotation speeds and corresponding K–L plots (**d**); (**e**) long-term stability tests; and (**f**) tolerance to alcohol poisoning tests of DBD-FeCo@NC and 20 wt.% Pt/C via the oxygen reduction reaction (ORR) cathodic current-time (*i*-*t*) method.

The catalytic performance of ORR was tested using RDE with the electrolyte of 0.1 M KOH. LSV curves (Figure 4a) for the FeCo@NC, DBD-FeCo@NC, and 20 wt% Pt/C were examined at a 10 mV/s scanning rate 1600 rpm rotation speed in O_2_-saturated electrolytes. Not surprisingly, DBD-FeCo@NC disclosed the limited current density of 5.66 mA/cm^2^ and an onset potential of 0.96 V vs. RHE, which was greater than FeCo@NC (4.42 mA/cm^2^, 0.88 V vs. RHE) and 20 wt.% Pt/C (5.01 mA/cm^2^, 0.93 V vs. RHE). It was observed that DBD-FeCo@NC showed the half-wave potential at 0.88 V vs. RHE, which was 100 mV higher than that of FeCo@NC without plasma treatment. The ORR performance was comparable to other alloy catalysts (Table 2). Figure 4b displayed that CV showed a noticeable reduction peak at a 50 mV s^−1^ scanning rate in O_2_-saturated electrolytes, but no peak in N_2_-saturated electrolytes. These results indicated the obvious ORR activity toward DBD-FeCo@NC. The reaction kinetics of DBD-FeCo@NC was measured by an ORR polarization technique at 625–2500 rpm (Figure 4c). The Koutecky–Levich (K–L) graphs of the DBD-FeCo@NC exhibited an approximately linear relationship between ω^−1/2^ and j^−1^ in Figure 4d. In the range of 0.3–0.6 V, the average electron transfer number n value for DBD-FeCo@NC was calculated to be about 3.8 from K–L graphs, indexing a near four-electron transfer mechanism. Durability tests were performed for 20 wt% Pt/C and DBD-FeCo@NC by *i*-*t* measurement (Figure 4e). After 32,000 s of continuous operation, the current density of DBD-FeCo@NC can still be maintained at 75.6% (vs 35.6%, 20 wt% Pt/C), manifesting that DBD-FeCo@NC exhibited good durability. The methanol tolerance of DBD-FeCo@ NC and 20 wt% Pt/C was examined by adding 3 M methanol to 0.1 M KOH solution after 200 s. The chronoamperometric response (Figure 4f) showed that DBD-FeCo@NC only decreased slightly, and its current remained at 89.2% after 1200 s, while the 20 wt% Pt/C was only 43.1% of its maximum current. The results displayed that the stability and selectivity of the DBD-FeCo@ NC catalyst for ORR was higher than 20 wt% Pt/C.

**Figure 5:**
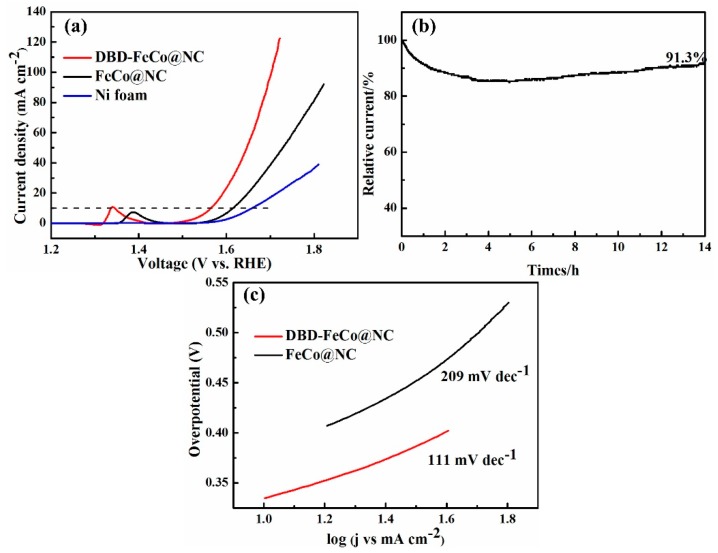
(**a**) The LSV polarization curves and (**b**) Tafel slope of OER for FeCo@NC and DBD-FeCo@NC, (**c**) Galvanostatic measurement of OER of DBD-FeCo@NC at a current density of 10 mA cm^−2^.

The electrocatalytic properties of FeCo@NC and DBD-FeCo@NC for the OER activity were also studied. As displayed in Figure 5a, the initial potential of DBD-FeCo@NC was 1.49 V vs. RHE, which was more negative than that of FeCo@NC (1.55 V vs. RHE), and the initial potential of nickel foam displayed 1.58 V vs. RHE, suggesting an enhanced OER activity in DBD-FeCo@NC. It is well known that the potential demanded to provide the current density of 10 mA/cm^2^ is the key benchmark for OER [43,44,45]. Under the current density of 10.0 mA/cm^2^, overpotential of 386 mV and 335 mV were estimated for FeCo@NC and DBD-FeCo@NC, respectively. As illustrated in Figure 5b, the DBD-FeCo@NC displayed a Tafel slope (111 mV/dec), which was much less than that of FeCo@NC (209 mV/dec), demonstrating the most favorable OER kinetics and highly active DBD-FeCo@NC. The current *i*-*t* curve (Figure 5c) showed excellent stability of DBD-FeCo@NC under a current density of 10 mA/cm^2^, which retained 91.3% of the initial catalytic current after 14 h of continuous testing.

Based on the structure and morphology of the catalysts, the excellent electrocatalytic activities for both ORR and OER can be attributed to several factors: (1) The active sites of Fe/Co-N_x_-C can adjust the electronic polarities and surface properties, which can improve the activity of the catalyst; (2) the unique core-shell structure offered more active sites and effectively prevented the coagulation and dissolution/redeposition of FeCo alloy nanoparticles, thus maintaining high electrocatalytic stability; (3) the plasma treatment made the catalyst possess more defect sites, which played an important role in improving catalytic performance; (4) the Co-Fe system enhanced the conductivity of the catalysts.

## 4. Conclusions

In summary, we synthesized N-doped graphite carbon-coated FeCo alloy core-shell nanoparticles via the microwave-assisted carbon bath method and further treatment with DBD plasma as bi-functional ORR/OER catalysts. The materials exhibited unique core-shell structure, Fe/Co-*N*-C sites, the existence of FeCo alloys, and enriched defect active sites. Because of these characteristics, DBD-FeCo@NC displayed excellent ORR performance, as well as good OER activity. This synthetic route is facile and scalable to prepare catalytic materials with different metal-doped and abundant defect active sites, giving a wide possibility for large-scale application in practice.

## Figures and Tables

**Table 1:** Atomic content of FeCo@NC and DBD-FeCo@NC.

Sample	Content (at.%)					Content of N Species (at.%)			
	C	O	N	Fe	Co	Pyridinic	Pyrrolic	Graphitic	Oxidized
FeCo@NC	50.42	32.89	1.8	9.44	5.45	0.26	0.30	0.26	0.98
DBD-FeCo@NC	60.05	24.65	1.67	8.64	4.99	0.58	0.33	0.26	0.50

**Table 2:** FeCo@NC and DBD-FeCo@NC compared with other alloy catalysts. Reversible hydrogen electrode (RHE); microwave-assisted carbon bath method (MW-CBM).

Catalysts	Preparation Method	Onset Potential (V vs. RHE)	Half-Wave Potential (V vs. RHE)	Limiting-Current Density (mA/cm^2^)	Ref.
PtNi/C	Solution synthesis	-	0.88	-	[16]
PtFe alloy	Solution synthesis	0.95	0.88	5.83	[17]
FeCo@NC-750	Furnace heating	0.94	0.80	4.82	[20]
FeNi@NCNTs	Furnace heating	0.95	0.77	4.70	[21]
FeCo@NC	MW-CBM	0.88	0.78	4.42	this work
DBD-FeCo@NC	MW-CBM	0.96	0.88	5.66	this work
